# Divergent SATB1 expression across human life span and tissue compartments

**DOI:** 10.1111/imcb.12233

**Published:** 2019-02-25

**Authors:** Simone Nüssing, Hui‐Fern Koay, Sneha Sant, Thomas Loudovaris, Stuart I Mannering, Martha Lappas, Yves d′Udekem, Igor E Konstantinov, Stuart P Berzins, Guus F Rimmelzwaan, Stephen J Turner, E Bridie Clemens, Dale I Godfrey, Thi HO Nguyen, Katherine Kedzierska

**Affiliations:** ^1^ Department of Microbiology and Immunology University of Melbourne Peter Doherty Institute for Infection and Immunity Parkville VIC Australia; ^2^ Australian Research Council Centre of Excellence for Advanced Molecular Imaging at the University of Melbourne Parkville VIC Australia; ^3^ Immunology and Diabetes Unit St Vincent's Institute of Medical Research Fitzroy VIC Australia; ^4^ Department of Medicine University of Melbourne St Vincent′s Hospital Fitzroy VIC Australia; ^5^ Obstetrics, Nutrition and Endocrinology Group Department of Obstetrics & Gynaecology University of Melbourne Mercy Hospital for Women Heidelberg VIC Australia; ^6^ Department of Cardiothoracic Surgery Royal Children′s Hospital and Melbourne Children′s Centre for Cardiovascular Genomics and Regenerative Medicine Parkville VIC Australia; ^7^ School of Health and Life Sciences Federation University Australia Ballarat VIC Australia; ^8^ Fiona Elsey Cancer Research Institute Ballarat VIC Australia; ^9^ Department of Viroscience Erasmus Medical Centre Rotterdam The Netherlands; ^10^ Center for Emerging Infections and Zoonoses University of Veterinary Medicine Hannover Germany; ^11^ Department of Microbiology Monash Biomedicine Discovery Institute Monash University Clayton VIC Australia

**Keywords:** Human CD8^+^ T cells, PD‐1, SATB1

## Abstract

Special AT‐rich binding protein‐1 (SATB1) is a global chromatin organizer capable of activating or repressing gene transcription in mice and humans. The role of SATB1 is pivotal for T‐cell development, with SATB1‐knockout mice being neonatally lethal, although the exact mechanism is unknown. Moreover, SATB1 is dysregulated in T‐cell lymphoma and proposed to suppress transcription of the *Pdcd1* gene, encoding the immune checkpoint programmed cell death protein 1 (PD‐1). Thus, SATB1 expression in T‐cell subsets across different tissue compartments in humans is of potential importance for targeting PD‐1. Here, we comprehensively analyzed SATB1 expression across different human tissues and immune compartments by flow cytometry and correlated this with PD‐1 expression. We investigated SATB1 protein levels in pediatric and adult donors and assessed expression dynamics of this chromatin organizer across different immune cell subsets in human organs, as well as in antigen‐specific T cells directed against acute and chronic viral infections. Our data demonstrate that SATB1 expression in humans is the highest in T‐cell progenitors in the thymus, and then becomes downregulated in mature T cells in the periphery. Importantly, SATB1 expression in peripheral mature T cells is not static and follows fine‐tuned expression dynamics, which appear to be tissue‐ and antigen‐dependent. Furthermore, SATB1 expression negatively correlates with PD‐1 expression in virus‐specific CD8^+^ T cells. Our study has implications for understanding the role of SATB1 in human health and disease and suggests an approach for modulating PD‐1 in T cells, highly relevant to human malignancies or chronic viral infections.

## Introduction

Special AT‐rich sequence‐binding protein 1 (SATB1) is a chromatin organizer and transcription factor^1^, ^2^ that gained increasing interest over recent years as a tumor marker. In human cancer studies, SATB1 is thought to be important in promoting tumor growth and invasion, including breast cancers,^3^ prostate cancers,^4^ colorectal cancers,^5^, ^6^ or pancreatic cancers.^7^ Dysregulated SATB1 expression was further linked to the pathogenesis of T‐cell lymphoma.^8^, ^9^

SATB1 originally was described to be expressed in T‐cell populations, especially in the thymus.^1^, ^2^, ^10^ Essential roles of SATB1 in organizing temporal and spatial expression of genes were shown for T‐cell development in murine thymi after the generation of SATB1‐*null* mice.^2^ SATB1‐*null* mice had small thymi and spleens and were fatal by the age of 3 weeks. Thymocyte development was blocked at the CD4^+^CD8^+^ double‐positive (DP) stage as only a few CD4^+^ and CD8^+^ single‐positive T cells survive and migrate to the periphery in SATB1‐*null* mice.^2^ SATB1 is differentially expressed during thymocyte development and is downregulated in peripheral CD4^+^ T cells after thymic exit.^11^ Although SATB1 has been well‐described in the mouse thymus, less is known about its expression and role in human thymocytes and peripheral T‐cell subsets. Earlier studies have shown that SATB1 mRNA is predominantly expressed in mouse and human thymus,^1^ with lower levels found in the brain and mammary glands in mice.^10^ SATB1 transcripts have also been detected in human testis^1^ and in cell lines including Mink lung cells and Jurkat (human) T cells.^10^ SATB1 was further shown by whole transcriptome RNA‐Seq analysis to be downregulated in human blood CD4^+^ regulatory T cells (T_regs_) and by flow cytometry in mouse T_regs._ 12

The downregulation of SATB1 in T cells occurred in murine models of T‐cell exhaustion, in which mice were infected with lymphocytic choriomeningitis virus clone 13 to establish a chronic infection. Microarray data showed that SATB1 gene expression was downregulated in exhausted CD8^+^ T cells during chronic infection compared to naïve CD8^+^ T cells.^13^ Exhausted CD8^+^ T cells upregulate the immune checkpoint molecule, programmed cell death protein 1 (PD‐1, CD279), leading to an inhibitory T‐cell program when binding to its ligand PD‐L1, as commonly observed during human malignancies or chronic viral infections. In human clinical trials, novel antibody‐mediated immunotherapies aimed at blocking PD‐1 are currently being used in patients with chronic conditions such as solid tumors, including melanoma^14^, ^15^, ^16^, ^17^, ^18^ (reviewed in 19) and HIV patients on anti‐retroviral treatment (reviewed in 20). The remarkable success of immunotherapies targeting PD‐1 in certain cancers highlights the significance of reversing T‐cell exhaustion.^21^

A link among SATB1, PD‐1 and cancer was found in a recent study in mice and human samples by Stephen *et al*. 2017, demonstrating that SATB1 recruits the nucleosome remodeling deacetylase complex to the regulatory regions of the *Pdcd1* gene, encoding PD‐1, and thereby preventing its transcription early after CD8^+^ T‐cell activation.^22^ Furthermore, the addition of transforming growth factor β (TGF‐β), frequently found in the tumor environment, to human T‐cell cultures, resulted in impairment of TCR‐induced SATB1 expression and therefore concomitant increase of PD‐1 expression. This was consistent with CD8^+^CD45RA^−^ T cells isolated from human ovarian cancer and compared to blood T cells which exhibited lower SATB1 expression, with higher PD‐1 expression in tumor infiltrating cells than in the periphery.^22^

SATB1 expression across T‐cell subsets from different tissue compartments in humans might be of importance for targeting PD‐1 in the clinic. Here, we show a comprehensive analysis of SATB1 expression across immune compartments from different human tissues by flow cytometry and correlate this to PD‐1 expression. We investigated SATB1 protein levels in healthy human tissues obtained from pediatric and adult donors, including different immune cell subsets from various human organs. We also examined SATB1 in antigen‐specific T cells against acute and chronic viral infections, specifically toward the most prominent HLA‐A*02:01‐restricted epitope from influenza A virus (IAV) (HLA‐A*02:01‐M1_58–66_ (M1_58_))^23^, ^24^ and Epstein–Barr virus (HLA‐A*02:01‐BMLF1_280–288_ (GLC)).^25^ Our study has implications for further understanding of differential SATB1 expression in lymphocytes across healthy human tissues.

## Results

### High levels of SATB1 expression in human thymic T‐cell progenitors and downregulation in peripheral human pediatric T cells

Previous studies in SATB1‐*null* mice demonstrated an essential role of SATB1 in T‐cell development in murine thymi, with thymocyte development being mainly blocked at the CD4^+^CD8^+^ double positive stage, leading to a very limited number of single‐positive CD4^+^ and CD8^+^ T cells migrating to the periphery.^2^ Differential levels of SATB1 expression were measured across various thymocyte subsets in mice, followed by downregulation of SATB1 in peripheral CD4^+^ T cells after thymic exit.^11^ In the present study, we extended these findings to humans and investigated the kinetics of SATB1 expression across different human tissue compartments and ages.

**Figure 1:**
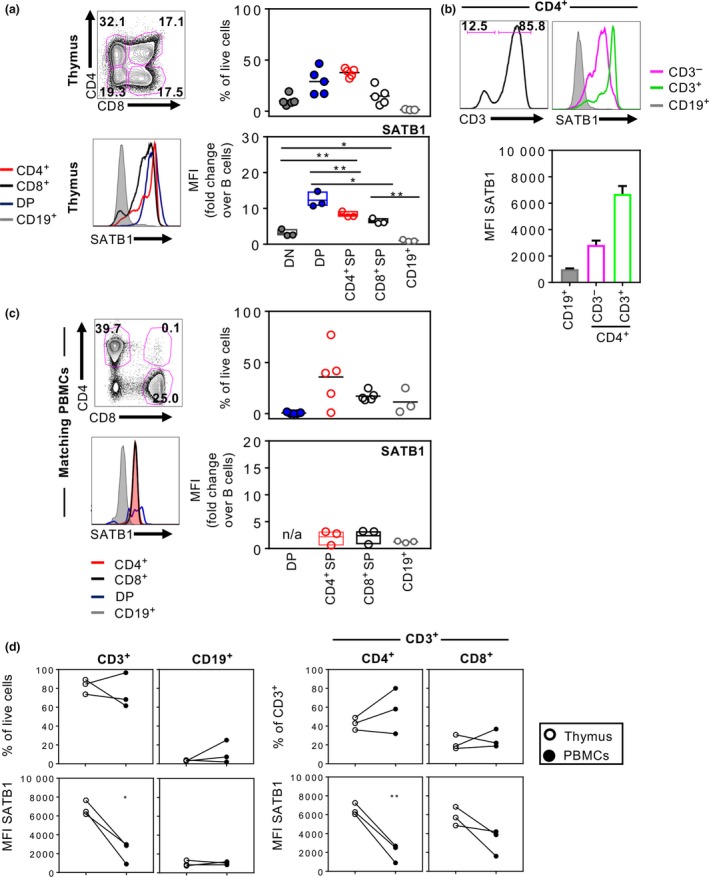
SATB1 expression in humans is downregulated from thymocyte precursors to peripheral T cells. Human thymocytes and PBMCs were immuno‐stained for flow cytometry to measure proportions of CD4^+^ SP, CD8^+^ SP, CD4^+^ CD8^+^ DP and CD4^−^ CD8^−^ DN T‐cell lineage subsets in human infant thymi (*n* = 5) **(a)** and matched pediatric PBMCs (*n* = 5) **(c)**. Representative histograms of SATB1 expression for each subset are shown alongside MFI‐fold change over thymic CD19^+^ B cells, which express low levels of SATB1 **(a** and **c)**. CD3 expression of CD4^+^ SP and MFIs of SATB1 are shown for CD3^−^ CD4^+^ and CD3^+^ CD4^+^ T‐cell progenitors in human thymus (*n* = 3) **(b)**. Comparisons of CD3^+^ T cell (including CD4^+^ and CD8^+^ subsets) and CD19^+^ B cell proportions and MFI levels of SATB1 expression between thymus and matching blood samples **(d)** were performed using one‐way ANOVA or Student's *t*‐test (*P ≤ 0.05; **P ≤ 0.01). Experiments were performed once for each donor within each tissue.

We utilized a rare cohort of matched human thymi and peripheral blood mononuclear cells (PBMCs) obtained from infants and young children aged 0–2 years (Supplementary table 1). Thymocytes were firstly analyzed according to their thymic T‐cell development subsets and showed highly abundant CD4^+^ single‐positive (CD4^+^ SP) (mean 37.7%, 32.1–42.2%) and CD4^+^CD8^+^ double‐positive cells (mean 28.9%, 16.6–46.5%), followed by CD8^+^ single‐positive (CD8^+^ SP) T‐cell progenitors (mean 14.9%, 5.8–28.1%), and to a lesser extent CD4^−^CD8^−^ double‐negative (DN) early T‐cell progenitors (mean 10.3%, 5.7–19.3%) (Figure 1a). As expected, CD3^−^CD19^+^ cells represented a rare thymic B cell compartment (mean 1.5%, 1.2–2.0%) (Figure 1a).

Thymic T‐cell progenitor subsets were intracellularly stained for SATB1 expression to define SATB1 protein levels among different thymocyte subsets (Figure 1a). SATB1 expression was the lowest in thymic CD19^+^ B cells (Figure 1a, grey histogram) in comparison to other thymic progenitor subsets, and thus was used to normalize geometric mean fluorescence intensity of SATB1 expression in T‐cell subsets across different experiments. Comparable to studies in mice,^11^ SATB1 expression was the highest in human DP cells (mean fold‐change of 12.3 over B cells) (Figure 1a, blue) and CD4^+^ SP cells (mean fold‐change of 8.2 over B cells) (Figure 1a, red) and lower in CD8^+^ SP T cells (mean fold‐change of 6.4 over B cells). CD4^+^ SP cells can be further divided into immature CD3^‐^CD4^+^ cells and CD3^+^CD4^+^ cells based on their CD3 expression. The majority of CD4^+^ SP cells were CD3^+^ cells (mean 92.4%), with higher mean fluorescence intensity levels of SATB1 compared to the immature CD3^−^CD4^+^ thymocytes (Figure 1b). Taken together, these findings provide the first evidence for the hierarchy of SATB1 expression in human thymocytes: DP CD4^+^CD8^+^ > CD4^+^ SP = CD8^+^ SP> DN CD4^−^CD8^−^ T cells.

To assess whether the downregulation of SATB1 occurs in mature human CD4^+^ and CD8^+^ T cells following thymic emigration, as previously shown for mice,^11^ SATB1 expression was measured in T‐cell subsets from matched pediatric PBMC samples, mainly consisting of CD4^+^ T cells (mean 35.8% of lymphocytes), followed by CD8^+^ T cells (mean 17.2%), with negligible numbers of CD4^+^CD8^+^ DP T cells (mean 0.6%) (Figure 1c). CD19^+^ B cells constituted a mean 11.5% of lymphocytes and, similarly to thymic B cells, expressed very low levels of SATB1 and thus were used to normalize SATB1 expression levels of T cells in PBMCs. Analysis of SATB1 in pediatric PBMCs showed that both CD4^+^ SP and CD8^+^ SP peripheral T cells had lower levels of SATB1 expression (mean fold‐change of 2.2 and 2.4 over B cells, respectively) compared to thymocytes (Figure 1c). Although the proportions of CD3^+^ T cells and CD19^+^ B cells of total live lymphocytes did not vary between matched thymus and pediatric PBMCs (Figure 1d), SATB1 expression in the peripheral CD4^+^ and CD8^+^ T‐cell compartments was reduced by almost fourfold in CD4^+^ T cells and threefold in CD8^+^ T cells when compared to mean fold‐changes in the thymus (Figure 1c, d). Therefore, in humans, SATB1 is downregulated in both CD4^+^ and CD8^+^ T‐cell compartments after exiting from the thymus.

### Reduced SATB1 expression in antigen‐experienced adult T cells in blood, spleen and lymph nodes

**Figure 2:**
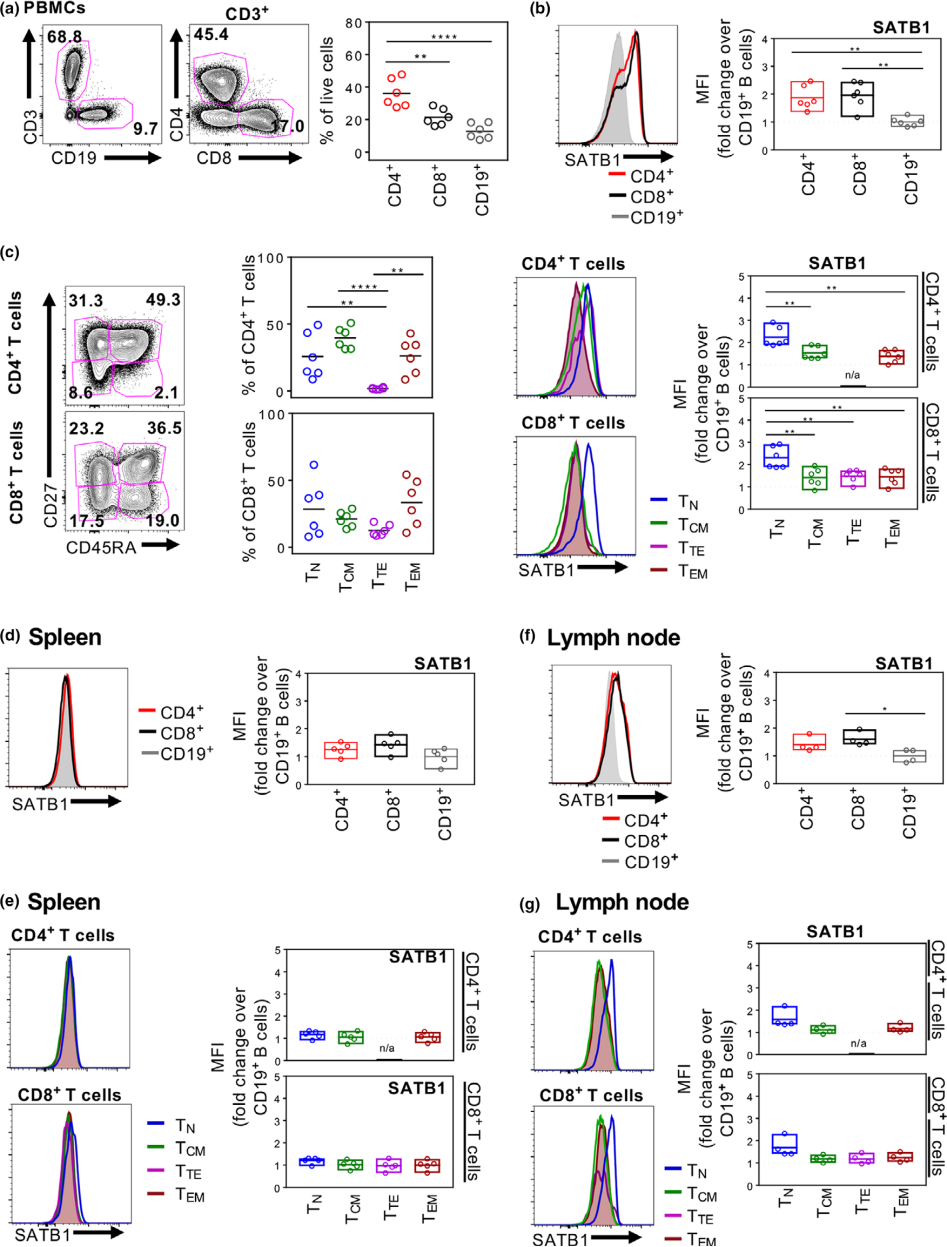
Diminished SATB1 expression in human spleen. Representative FACS plots and summarized CD4^+^, CD8^+^ CD3^+^ T cell and CD19^+^ B cell populations in adult PBMCs (*n* = 6) **(a)** and their respective SATB1 expression as MFI‐fold change over B cells in PBMCs **(b)** spleen (*n* = 5) **(d)** and lymph node (*n* = 4) **(f)**. CD27^+^ CD45RA ^+^, CD27^+^ CD45RA ^−^, CD27^−^ CD45RA ^+^, CD27^−^ CD45RA ^−^ T‐cell compartments of CD4^+^ and CD8^+^ T cells from adult PBMCs **(c)** with their respective SATB1 expression as representative histograms and MFI‐fold change over CD19^+^ B cells in PBMCs **(c)**, spleen **(e)** and lymph node **(g)**. One‐way ANOVA was performed between groups (*P ≤ 0.05; **P ≤ 0.01, ***P ≤ 0.001, ****P ≤ 0.0001). Experiments were performed once for each donor within each tissue.

As the data presented in Figure 1 were obtained from matched thymus/PBMC samples of pediatric donors up to 2 years of age, we further investigated SATB1 expression in T and B lymphocyte populations isolated from the blood (Figure 2a), spleen (Supplementary figure 1a) and mesenteric lymph nodes (Supplementary figure 1c) of adult donors (22–60 years, Supplementary table 1). Additionally, SATB1 expression was analyzed in CD4^+^ and CD8^+^ T cells according to their CD27 and CD45RA expression to differentiate between CD27^+^CD45RA^+^ naïve T cells (T_N_), CD27^+^CD45RA^−^ central memory T cells (T_CM_), CD27^−^CD45RA^−^ effector memory T cells (T_EM_) and CD27^−^CD45RA^+^ terminal effector T cells (T_TE_) (Figure 2c, Supplementary figure 1b and d). Similar to pediatric PBMCs, adult PBMCs primarily consisted of CD4^+^ T cells (mean of 36.2% of live lymphocytes), followed by CD8^+^ T cells (mean of 21.5%) and then B cells (mean of 12.8%). In alignment with pediatric thymocytes and PBMCs, there were no differences in SATB1 mean fluorescence intensity values between CD4^+^ and CD8^+^ T cells in adult PBMCs, but there was a modest yet significant increase in SATB1 expression levels in both CD4^+^ and CD8^+^ T cells when comparing against CD19^+^ B cells (mean fold‐changes of 1.9 and 2.0 over B cells, respectively) (Figure 2b). Interestingly, we observed significant differences in SATB1 expression levels between T_N_ cells and antigen‐experienced T_CM_, T_TE_ and T_EM_ subsets for both CD4^+^ T cells (T_N_ 2.2, T_CM_ 1.5, T_TE_ n/a and T_EM_ 1.4; mean fold‐change over B cells) and CD8^+^ T cells (T_N_ 2.3, T_CM_ 1.4, T_TE_ 1.5 and T_EM_ 1.5; mean fold‐change over B cells) (Figure 2c, right panels). Our results reveal that, in addition to SATB1 downregulation in T cells exiting the thymus, there is a hierarchy in SATB1 expression in mature peripheral blood adult CD4^+^ and CD8^+^ T cells, with SATB1 expression being significantly higher in naïve T cells compared to antigen experienced effector/memory‐like T cells.

As a next step, we assessed whether SATB1 was further downregulated following migration of T cells from the blood to secondary lymphoid tissues such as the spleen (Figure 2d, e) and lymph nodes (Figure 2f, g). SATB1 expression ratios were marginally lower in splenocytes compared to PBMCs, with mean fold‐changes of 1.3 and 1.4 in splenocyte CD4^+^ T cells and CD8^+^ T cells, respectively, over B cells. Strikingly, there were no significant differences in SATB1 expression between T_N_ and effector/memory‐like T cells for both CD4^+^ and CD8^+^ T cells in the spleen.

Given SATB1's differential expression between T_N_ and effector/memory‐like T cells in blood, but not spleen, SATB1 levels were next investigated in lymph nodes, at the site of antigen encounter. Here, SATB1 expression ratios over B cells were moderately higher in CD4^+^ T cells (mean of 1.4) and CD8^+^ T cells (mean fold‐changes of 1.4 and 1.6, respectively), similar to spleen. However, similar to adult blood but not spleen, slight differences in SATB1 expression were observed between T_N_ and effector/memory‐like T cells (CD4^+^ T cells: T_N_ 1.6, T_CM_ 1.1, T_TE_ n/a, and T_EM_ 1.2; CD8^+^ T cells: T_N_ 1.7, T_CM_ 1.2, T_TE_ 1.2, and T_EM_ 1.2; mean fold‐change over B cells), suggesting that the downregulation of SATB1 expression in mature peripheral T‐cell subsets was tissue‐dependent and perhaps related to anatomical sites of antigen encounters.

### High SATB1 expression in T_N_ and T_CM_ within human cord blood

**Figure 3:**
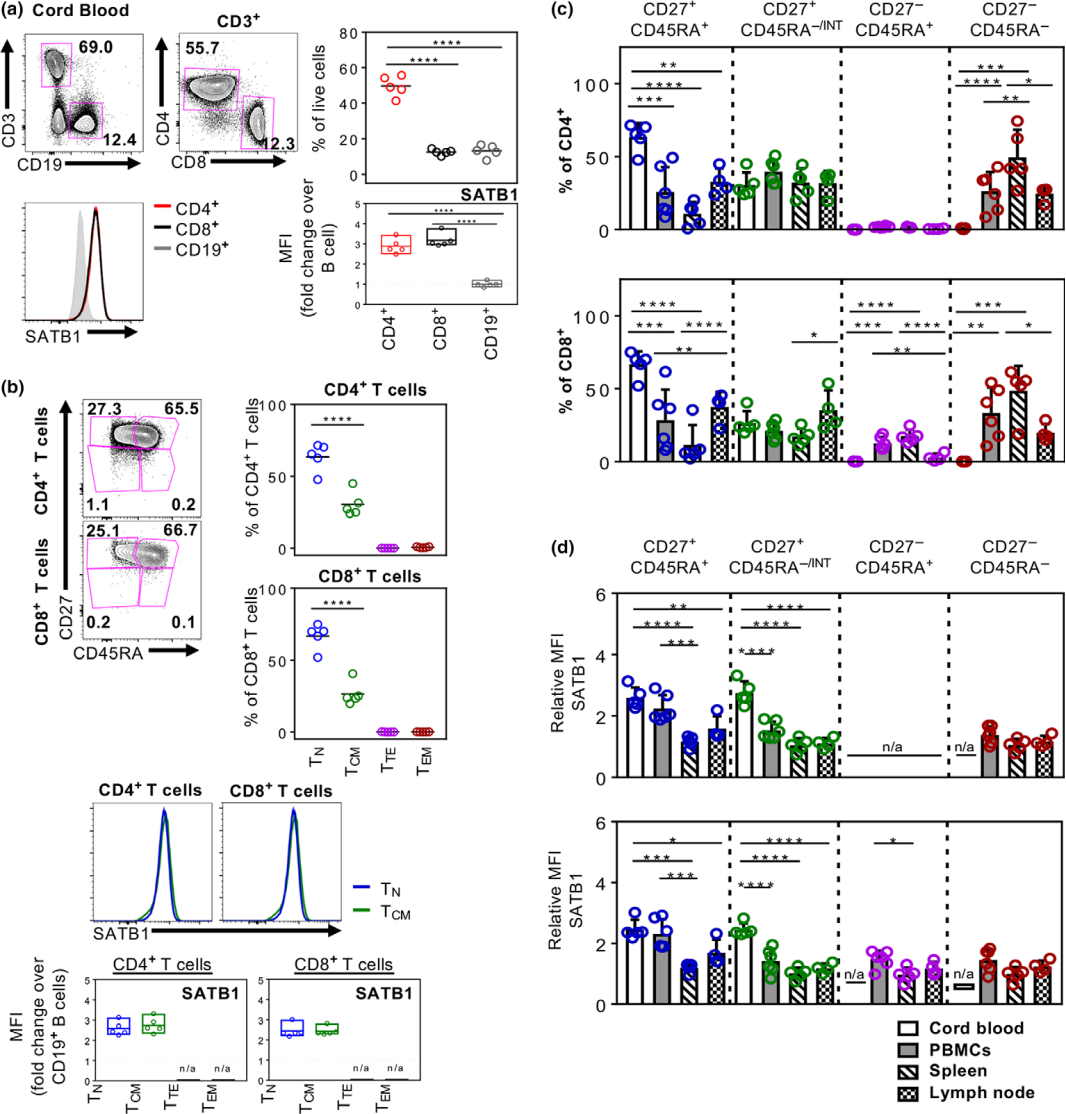
SATB1 is highly expressed within T cells in human cord blood. SATB1 expression in CD4^+^, CD8^+^ CD3^+^ T cell and CD19^+^ B cell populations in human cord blood (*n* = 5) **(a)** and within CD27^+^ CD45RA ^+^, CD27^+^ CD45RA ^−^, CD27^−^ CD45RA ^+^, CD27^−^ CD45RA ^−^ T cell compartments of CD4^+^ and CD8^+^ T cells **(b)**, as representative histograms and MFI‐fold change over CD19^+^ B cells (bottom panels). Summarized proportions of CD27^+^ CD45RA ^+^, CD27^+^ CD45RA ^−/^ ^INT^, CD27^−^ CD45RA ^+^, CD27^−^ CD45RA ^−^ T cell compartments of CD4^+^ and CD8^+^ T cells across different human tissues **(c)** with their respective SATB1 expression as MFI‐fold change over CD19^+^ B cells **(d)**. One‐way ANOVA analysis was performed (*P ≤ 0.05; **P ≤ 0.01, ***P ≤ 0.001, ****P ≤ 0.0001). Experiments were performed once for each donor within each tissue.

To further explore whether the observed differential expression of SATB1 between T_N_ and effector/memory‐like T cells occurred predominantly as a result of prior antigenic exposure, we assessed SATB1 levels in CD4^+^ and CD8^+^ T cells in human cord blood, which are essentially “naïve” from external pathogens. Strikingly, we found significantly higher levels of SATB1 expression in both CD4^+^ and CD8^+^ T cells, with a mean fold‐change of 2.9 increase in CD4^+^ T cells and 3.2‐fold increase in CD8^+^ T cells, over B cells in human cord blood (Figure 3a), which were approximately twofold higher than the changes observed in adult blood and lymph nodes. As expected, the majority of T cells in human cord blood were naïve, with means of 63.5% and 66.7% of CD4^+^ and CD8^+^ T cells, respectively, displaying a CD27^+^CD45RA^+^ T_N_ phenotype, while the remaining CD27^+^ cells were intermediate in their CD45RA expression (Figure 3b), but they did not fall into distinct effector/memory‐like phenotypes observed in adult blood and tissues. Nevertheless, SATB1 expression levels were comparable between the cord blood T_N_ and CD27^+^CD45RA^−/INT^ T‐cell subsets, highlighting that the downregulation of SATB1 expression between naïve and antigen‐experienced T cells in human peripheral blood and lymph nodes might be a consequence of antigenic encounters during human life.

Overall, our study across different human tissues and ages confirms that the naïve CD4^+^ and CD8^+^ T‐cell compartment is the largest in cord blood and the lowest in spleen, which harbors the largest T_EM_ reservoir among all the tissues analyzed (Figure 3c). The greatest differential SATB1 expression ratios over B cells were observed in naïve T cells with further tissue‐specific SATB1 downregulation in the spleen and lymph nodes compared to cord and adult blood (Figure 3d). These results show that SATB1 expression is not only dependent on antigen experience but can also be influenced by tissue‐specific localization of CD4^+^ and CD8^+^ T cells.

### Discordant expression of SATB1 and PD‐1 in virus‐specific CD8^+^ T cells

To further understand whether the observed downregulation of SATB1 expression in T cells occurs following antigenic encounters, we analyzed antigen‐specific CD8^+^ T cells present in human adult blood for two immunodominant HLA‐A*02:01‐restricted epitopes, influenza‐derived M1_58–66_ (GILGFVFTL; referred to as A2/M1_58_) and EBV‐derived BMLF1_280–288_ (GLCTLVAML; referred to as A2/GLC). IAV‐ and EBV‐specific CD8^+^ T cells were enriched from four HLA‐A*02:01‐positive donors using A2/M1_58_‐ and A2/GLC‐tetramers using the well‐established tetramer‐associated magnetic enrichment approach.^23^, ^24^, ^25^ Enriched tetramer‐positive CD8^+^ T cells were then analyzed for T‐cell differentiation phenotype and co‐expression of SATB1 and the immune checkpoint protein PD‐1, shown to be pivotal in T‐cell exhaustion following chronic infections or cancer and regulated by SATB1.^22^

**Figure 4:**
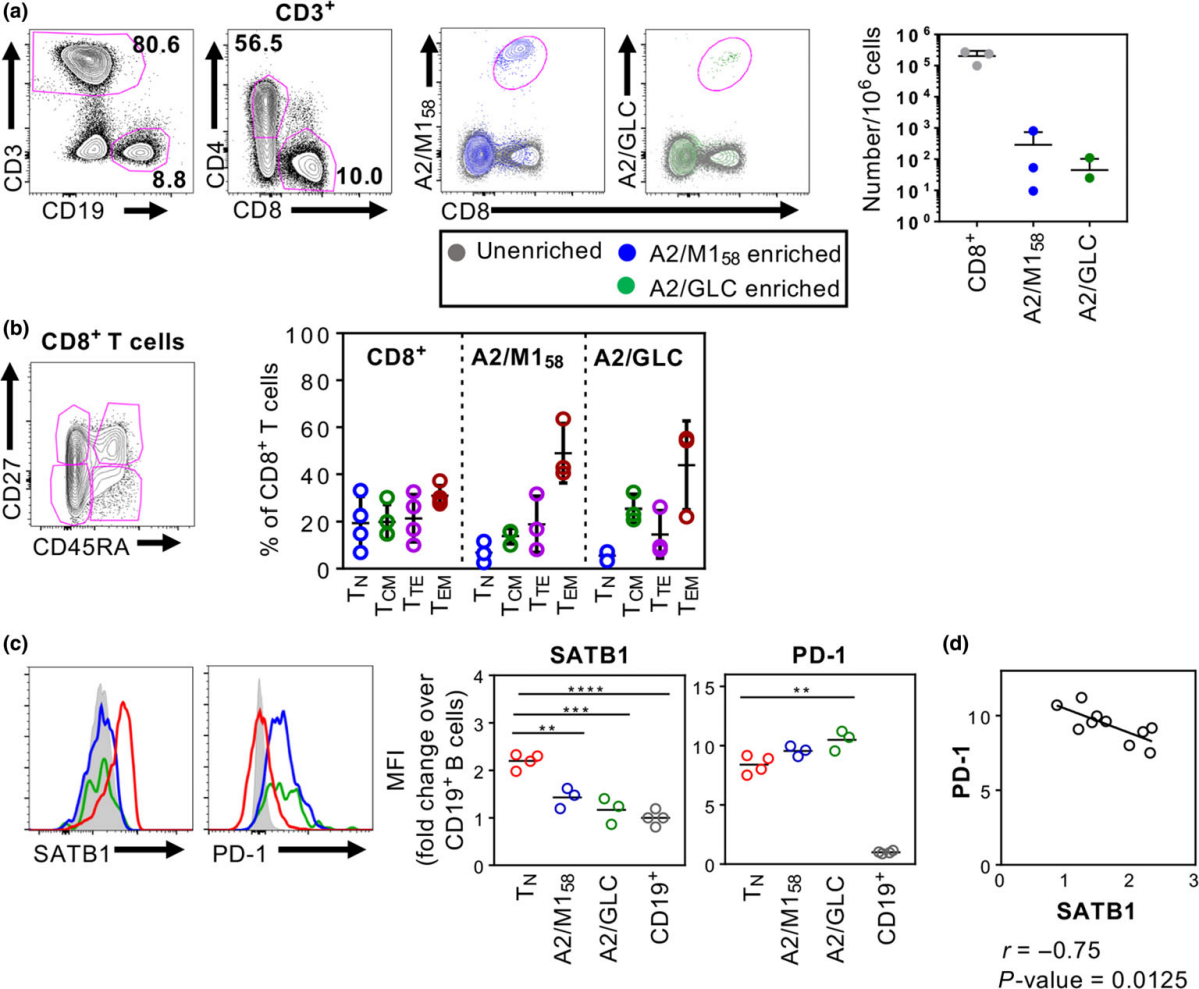
SATB1 expression is downregulated in antigen‐specific CD8^+^ T cells. Representative FACS plots of CD3^+^ T cell and CD19^+^ B cell populations, and CD4^+^ and CD8^+^ T cell populations from human PBMCs (*n* = 4), followed by overlay FACS plots of unenriched and enriched A2/M1‐ and A2/GLC‐specific CD8^+^ T cells, and summarized CD8^+^ T cell (*n* = 4) and tetramer^+^ frequencies (*n* = 3) per 10^6^ total live lymphocytes **(a)**. CD27 and CD45RA quadrant expression in unenriched total CD8^+^ T cells and enriched A2/M1‐ and A2/GLC‐specific CD8^+^ T cells **(b)**. SATB1 and PD1 expression levels of unenriched CD8^+^ CD27^+^ CD45RA ^+^ T_N_ were compared to enriched A2/M1‐ and A2/GLC‐specific CD8^+^ T cells, normalized to B cells **(c)**. Pearson correlation of PD‐1 *versus* SATB1 of combined data points from unenriched CD8^+^ CD27^+^ CD45RA ^+^and enriched A2/M1‐ and A2/GLC‐specific CD8^+^ T cells **(d)**. One‐way ANOVA analysis or Pearson correlation was performed (**P ≤ 0.01, ***P ≤ 0.001, ****P ≤ 0.0001). Experiments were performed once for each donor within each tissue.

Data from four donors showed tetramer‐positive CD8^+^ T populations with different mean precursor frequencies, specific for either IAV‐M1 and/or EBV‐GLC epitopes following tetramer‐associated magnetic enrichment (Figure 4a). Enriched A2/M1_58_‐ and A2/GLC‐specific CD8^+^ T cells were predominantly CD27^−^CD45RA^−^ T_EM_ cells (Figure 4b). SATB1 expression was compared between acute (IAV‐A2/M1_58_) and chronic (EBV‐A2/GLC) antigen‐specific CD8^+^ T cells from the enriched fraction, to naïve CD8^+^ T_N_ cells from the unenriched fraction, which were normalized to SATB1 expression levels in B cells from the unenriched fractions (Figure 4c). There was a significant reduction in SATB1 ratios between T_N_ (mean fold‐change of 2.2) and IAV‐A2/M1_58_‐specific CD8^+^ T cells (mean fold‐change of 1.43), and between T_N_ and EBV‐A2/ GLC‐specific CD8^+^ T cells (mean fold‐change of 1.17) (Figure 4c). As PD‐1 can be overexpressed in conditional SATB1‐cKO mice^22^ as well as during chronic viral infections,^26^, ^27^ we also examined PD‐1 expression and found that PD‐1 expression was highest in antigen‐experienced CD8^+^ T cells compared to naïve CD8^+^ T cells (EBV‐A2/GLC 9.7, IAV‐A2/M1_58_ 9.2 and T_N_ 8.1; mean fold‐change over B cells). Interestingly, discordant patterns of lower SATB1 expression and higher PD‐1 expression was more pronounced in antigen‐specific CD8^+^ T cells toward the chronic EBV‐A2/GLC epitope compared to the acute IAV‐A2/M1_58_ epitope. However, when all the antigen‐specific CD8^+^ T cell data points were pooled together, there was a significant negative correlation between PD‐1 and SATB1 expression (Figure 4d), thus indicating a potential role of SATB1 in regulating PD1 expression in virus‐specific CD8^+^ T cells *ex vivo* accordant to previous cancer studies.

### Re‐inducible SATB1 expression in CD8^+^ T_CMs_ after TCR‐mediated stimulation

Given the differential SATB1 expression observed between T_N_ and antigen‐experienced T cells, we sought to define when SATB1 undergoes downregulation following the antigenic encounter, and whether downregulation of SATB1 in antigen‐experienced T cells was static or re‐inducible and therefore can be upregulated again after re‐stimulation. Previously, activation of human CD4^+^ and CD8^+^ T cells via co‐stimulation of the CD3/CD28 pathway was showed to upregulate SATB1, as detected by western blot and qPCR.^22^, ^28^ Similarly, SATB1 expression increases with TCR signaling in murine T cells from spleen^22^ and in the thymus.^11^ However, these studies did not differentiate between T_N_ and antigen‐experienced T cells, which as shown by our present study, differ greatly with respect to SATB1 expression.

**Figure 5:**
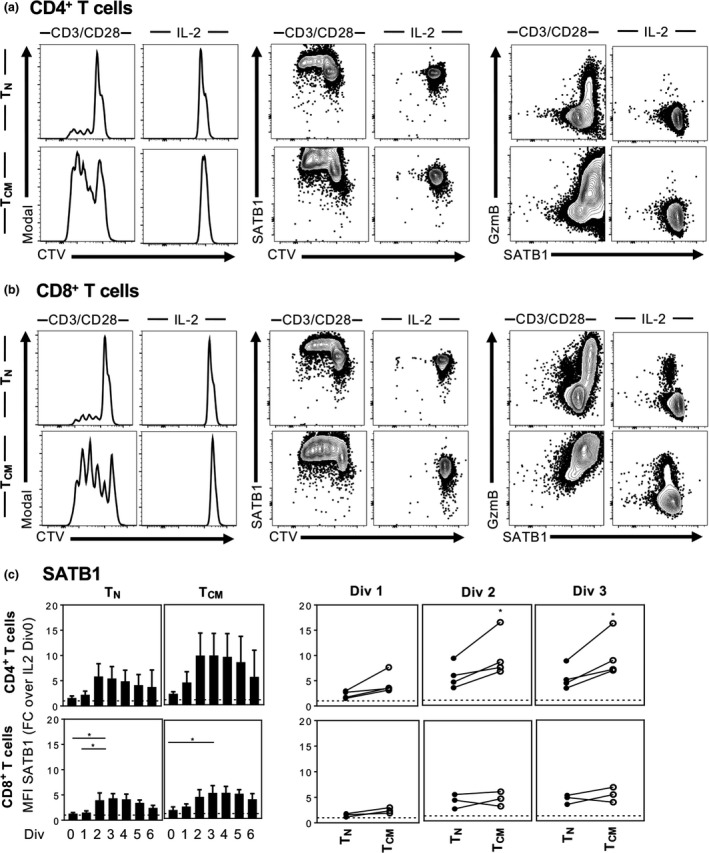
Temporal SATB1 expression following TCR‐mediated stimulation and cell division. Representative CTV‐histograms and FACS plots of CTV and GzmB *versus* SATB1 of CD27^+^ CD45RA ^+^ naïve and CD27^+^ CD45RA ^−^ T_CM_ CD4^+^ (*n* = 4) **(a)** and CD8^+^ T cells (*n* = 3) **(b)** sorted from PBMCs and *in vitro* stimulated for 4 days with anti‐CD3/anti‐CD28/IL‐2 (left panels) or IL‐2 alone (right panels) **(a** and **b)**. Summarized MFI of SATB1 expression per cell division (Div) in CD27^+^ CD45RA ^+^ naïve and CD27^+^ CD45RA ^−^ T_CM_ CD4^+^ **(c**, top panels**)** and CD8^+^ T cells (**c**, bottom panels) following TCR‐mediated stimulation is shown as fold change over IL‐2 alone control in division zero **(c**, Div 0**)**. One‐way ANOVA or Student′s *t*‐test was performed between groups (*P ≤ 0.05). Experiments were performed once for each donor within each tissue.

To measure the dynamics of SATB1 protein levels following TCR‐stimulation, naïve T_N_ cells (high SATB1) and antigen‐experienced CD8^+^ T_CM_ cells (low SATB1) were bulk‐sorted from human PBMCs before labeling with cell trace violet and then stimulated *in vitro* with anti‐CD3/anti‐CD28^22^, ^28^ in the presence of IL‐2. SATB1 expression was assessed on day 4 according to cell division and compared to unstimulated cells treated with IL‐2 alone. Histogram peaks of cell division based on the reduction in cell trace violet intensity showed that in both CD4^+^ (Figure 5a, left panels) and CD8^+^ T‐cell populations (Figure 5b, left panels), T_CM_ cells had a greater proliferative capacity compared to T_N_ cells, following TCR stimulation. Interestingly, within both CD4^+^ (Figure 5a, middle panels) and CD8^+^ T‐cell populations, albeit to a lesser extent (Figure 5b, middle panels), SATB1 expression, measured per division as a fold‐change over undivided cells in the unstimulated group, was upregulated in T_N_ cells (CD4^+^ 5.9, CD8^+^ 3.9; mean fold‐change over undivided), and even higher in T_CM_ cells (CD4^+^ 9.8, CD8^+^ 4.4; mean fold‐change over undivided), when cells had divided at least twice compared to the undivided cells left with IL‐2 only (Figure 5c). Additionally, analysis of GzmB expression, as an effector CD8^+^ T cell molecule, together with SATB1 expression, demonstrated that T cells with higher GzmB expression also displayed higher SATB1 levels, as compared to T cells not producing GzmB (Figure 5a, b, right panels).

Temporal changes in SATB1 expression was most apparent in CD4^+^ T cells, where SATB1 expression was elevated early on within the first two divisions in T_N_ cells and T_CM_ cells, which was then downregulated in later divisions (Figure 5c). It is also important to note that CD4^+^ T_CM_ starting with lower SATB1 expression *ex vivo* (data shown in Figure 2) showed higher SATB1 upregulation potential following TCR stimulation, as compared to T_N_ cells which started with higher baselines of SATB1 expression, suggesting a maximum threshold and/or saturation level of SATB1 expression in T cells (Figure 5c). These data provide further evidence for differential SATB1 expression dynamics between T_N_ and T_CM_ in humans, especially for CD4^+^ T cells.

Taken together, our results show that SATB1 expression in humans is the highest in T cell progenitors in the thymus, and then becomes downregulated in T cells which egress from the thymus. Importantly, SATB1 expression in peripheral mature T cells is not static and follows fine‐tuned expression dynamics, which appear to be tissue‐ and antigen‐dependent.

### Differential SATB1 expression across human organs

**Figure 6:**
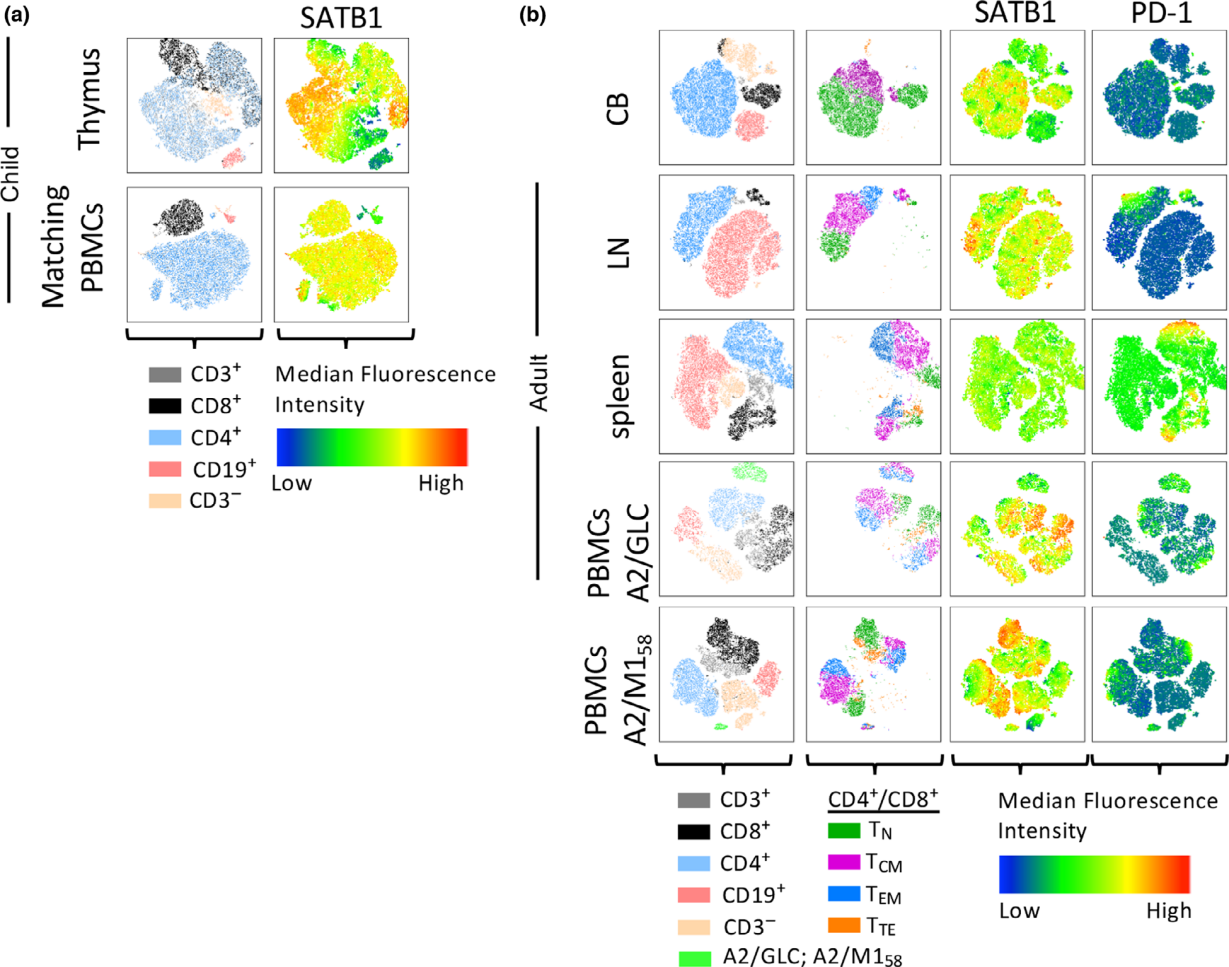
SATB1 expression across age and different human tissues. Representative t‐SNE analysis of thymus and matching PBMCs from a child **(a)**, cord blood, adult LN, spleen, and PBMCs **(b)**. Samples were gated on CD3^−^ (peach), CD3^+^(grey), CD19^+^ (rose), CD4^+^ (light blue), and CD8^+^ (black) populations. CD4^+^ and CD8^+^ T‐cell populations in **(b)** were further analyzed into CD27^+^ CD45RA ^+^ (dark green), CD27^+^ CD45RA ^−^ (violet), CD27^−^ CD45RA ^+^ (dark blue) and CD27^−^ CD45RA ^−^ (orange) populations. Median fluorescence intensity is displayed for SATB1 **(a)** and PD‐1 expression **(a** and **b)** after live/lymphocyte‐gated events were down sampled to 25 000 events using the FlowJo software (10.5). For A2/M1 or A2/GLC‐enriched PBMCs samples, live/lymphocyte‐gated events of their respective unenriched sample was down‐sampled to 25 000 events and concatenated with the enriched tetramer^+^ population for t‐SNE analysis. Experiments were performed once for each donor within each tissue.

We comprehensively compared SATB1 expression in different CD27/CD45RA CD4^+^ and CD8^+^ T‐cell subsets within human blood and tissues for both pediatric and adult donors, as well as for antigen‐experienced CD8^+^ T cells, relative to SATB1 expression in B cells within each population which expresses relatively low levels of SATB1. We then sought to define the overall distribution of SATB1 across thymus, lymph nodes, spleens, PBMCs of cord blood, children and adult donors. Furthermore, we aimed to determine how SATB1 expression correlates to, or co‐localizes with, PD‐1 expression. We performed t‐distributed stochastic neighbor embedding (t‐SNE) analysis across all the different tissues to visualize the distribution of SATB1 expression in relation to PD‐1 expression in different lymphocyte populations (Figure 6).

T‐SNE data from one representative donor per tissue showed highly diverse expression patterns of SATB1 in human thymus during the different T progenitor developmental stages (Figure 6a). Conversely, SATB1 expression in matching PBMCs from this 1.5‐year‐old pediatric donor demonstrated homogeneous expression across all T‐cell populations, and very low levels found only in CD19^+^ B cells. Similarly, in human cord blood, SATB1 was mainly expressed in CD3^+^CD4^+^ or CD3^+^CD8^+^ T cells, but low in CD19^+^ B cells (Figure 6b). In contrast, the lymph nodes showed high levels of SATB1 in smaller clusters within the B cell population, which was not observed in the other tissues. In general, high SATB1 expression tended to co‐localize with high CD27 and CD45RA co‐expression. However, the patches of high SATB1 expression did not co‐localize with high PD‐1 expression. In alignment with our earlier analysis of SATB1 expression within the spleen (Figure 2d, e), t‐SNE analysis showed lowest SATB1 expression in spleen with no median fluorescence intensity differences of SATB1 across CD3^+^ T cell and CD19^+^ B cell compartments, in comparison to all the tissues analyzed. Interestingly, SATB1 expression was mostly reduced in the A2/M1_58_‐ and A2/GLC‐antigen‐specific CD8^+^ T‐cell islands in comparison to other T‐cell areas.

Overall, our study shows that SATB1 is not only important in the thymus as previously thought, but that it may also has an extended role in the periphery, being preferentially expressed in less differentiated T‐cell subsets in the blood (especially in young individuals) and lymph nodes, but not in the spleen. The exact consequences of  its differential expression in the periphery remain to be elucidated.

## Discussion

SATB1 has been shown to be dysregulated in human T‐cell lymphoma.^8^, ^9^ Given its role in regulating PD‐1,^22^ a T‐cell exhaustion marker,^13^ SATB1 could be an attractive molecule to understand PD‐1 regulation in current cancer immunotherapeutic approaches which aim to reverse T‐cell exhaustion by PD‐1 blockade. In this study, we have characterized the level of SATB1 expression in human T‐cell differentiation subsets across different human tissues in pediatric and adult donors. Our study is important for understanding common differential SATB1 expression patterns in healthy individuals. Given that SATB1 levels are dysregulated in various cancers, including lymphoma, with a further link to PD‐1 regulation, our study can be of relevance to anti‐PD‐1 immunotherapies in cancer clinical trials.

Here, we comprehensively monitored SATB1 expression over various lymphocyte subsets across different human lymphoid organs. Aligning with previous studies in mice,^11^ our findings verify and greatly extend the importance of SATB1 beyond the thymus in humans. In addition to demonstrating high SATB1 expression levels in human thymus, we also show that the expression was differentially distributed across distinct T‐cell lineage subsets, with the highest SATB1 protein expression levels detected in immature CD4^+^CD8^+^ DP thymocytes, followed by CD4^+^ SP then CD8^+^ SP thymocytes, and subsequent downregulation in mature peripheral blood CD4^+^ and CD8^+^ T cells which exit the thymus.

To date, murine studies have focused on the role of SATB1 in T‐cell lineage studies in the thymus and spleen using crude flow cytometry, western blot and northern blot methods. There is also a paucity of evidence describing SATB1 expression within human lymphocytes, with only a few reports analyzing bulk populations of T cells in human cord blood and adult PBMCs.^10^, ^12^, ^22^, ^28^ In a seminal study by Stephen *et al.*,^22^ SATB1 expression via Western blotting was compared in non‐activated *versus* activated human CD4^+^ and CD8^+^ T cells, via the CD3/CD28 TCR stimulation pathway, in the presence or absence of TGF‐β. There was a general upregulation of SATB1 expression after TCR‐stimulation which was impeded by the presence of TGF‐β. In support, Ahlfors *et al*.^28^ showed via Western blot and qPCR that SATB1 was upregulated in CD4^+^ T cells from human cord blood following TCR stimulation with anti‐CD3/anti‐CD28 in the presence of the T‐helper 2 cytokine IL‐4. Furthermore, studies in mice have also shown increases in SATB1 expression in T cells from spleen^22^ and thymus^11^ following TCR‐mediated activation. However, these studies did not define T cells further into their differentiation states of naïve and antigen‐experienced T cells, whereas our analysis has shown that there are temporal changes in SATB1 expression with cell division between naïve and effector/memory T cells following TCR stimulation. However, it is unclear whether increased resting levels of SATB1 interfere with T_N_ cells to induce rapid proliferation or whether T_CM_ cells have a greater capacity to proliferate due to lower SATB1 levels, or whether it is the different expression dynamics of SATB1 that play a role in T‐cell proliferative capacity and/or effector function (i.e. expression of granzyme B).

In peripheral tissues, the dysregulation of SATB1 expression is involved in various human cancers. Tumor stage‐dependent downregulation of SATB1 by miR‐155 was reported in cutaneous T‐cell lymphomas.^8^ Overexpression of SATB1 has been reported in cutaneous anaplastic lymphoma driven by the SATB1 promoter methylation state.^9^, ^29^ These contrasting findings suggest a complex role for SATB1 depending on the microenvironment and hence a greater level of understanding is needed surrounding the normal expression of the chromatin organizer over different lymphocyte and tissue compartments before we can begin to understand the consequences of SATB1 dysregulation in human cancers. Here, we show that SATB1 is highly expressed in naïve cells and downregulated in antigen‐experienced CD8^+^ T cells, which could be related to the type of antigen, as we observed minor differences in SATB1 expression between IAV‐ and EBV‐specific CD8^+^ T cells. SATB1 expression may be additionally contingent on the tissue itself where the T cell resides, since differences observed in SATB1 expression in CD4^+^ and CD8^+^ T cells between adult human spleen and lymph nodes were found.

In murine studies, SATB1 was shown to act as a regulator of the *Pdcd1* gene^22^ encoding the immune checkpoint protein PD‐1 in CD8^+^ T cells, by binding to the *CR‐B* and *CR‐C* region, two key transcriptional enhancer sites of the *Pdcd1* gene.^30^ With 98% homology of SATB1 amino acid sequences between human and mice^31^ and similar negative correlation in PD‐1 expression upon downregulation of SATB1 in human CD8^+^ T cells, makes SATB1 a potential target for further downstream targeting in human malignancies. Here, we show a potential negative correlation of SATB1 and PD‐1 expression over different human tissues, making it more relevant in combination for treatment in SATB1‐dysregulated human malignancies.

Taken together, our study shows, at a great level of resolution, that SATB1 expression over different human tissues naturally undergoes fine‐tuned differential expression levels and likely decreases from infants to adults, such as from CD27^+^CD45RA^+^ naïve T cells to IAV‐ and EBV‐antigen experienced CD8^+^ T cells. Our findings can inform studies addressing PD‐1 regulation by SATB1 and thus are relevant to human malignancies or chronic viral infections.

## Methods

### Human blood and tissue samples

Young human thymi and matching peripheral human blood samples (age 0–2 years, Supplementary table 1) were obtained from the Royal Children's Hospital (Victoria, Australia). Human umbilical cord blood was obtained from the Mercy Hospital for Women (Victoria, Australia). Buffy packs of healthy adult donors (mean age 48.3 years, range 22–60) were obtained from the Australian Red Cross Blood Service (Victoria, Australia). Peripheral blood mononuclear cells were isolated by Ficoll‐Paque (GE Healthcare, Uppsala, Sweden) density‐gradient purification before cryopreservation. Spleen (mean age 48.6 years, range 33–68) and mesenteric/pancreatic lymph nodes (mean age 53.3 years, range 28–68) were obtained from deceased organ donors procured through DonateLife (Victoria, Australia) (Supplementary table 1). Human thymi were meshed through 70 μm cell strainers (Miltenyi Biotec, Bergisch Gladbach, Germany) into RPMI‐1640 (2 mm EDTA, ThermoFisher Scientific, Waltham, MA, USA) on ice. Spleens and lymph nodes were finely chopped and enzymatically digested with type III collagenase (1 mg mL^−1^, Worthington, Lakewood, NJ, USA) and DNase I (0.5 mg mL^−1^, Roche, Basel, Switzerland) in RPMI for 45–60 min (37°C/5% CO_2_) before meshing through 70 μm cell strainers. Splenic red blood cells were lysed with RBC lysis solution (0.168 m ammonium chloride (APS Chemicals Limited, Seven Hills, NSW, Australia), 0.01 mm EDTA, and 12 mm sodium bicarbonate (Sigma‐Aldrich, Darmstadt, Germany) in Baxter water (Baxter Healthcare, Old Toongabie, NSW, Australia). Isolated mononuclear cells were cryopreserved before use.

### Human ethics

Human experimental work was conducted according to the Declaration of Helsinki Principles and the Australian National Health and Medical Research Council (NHMRC) Code of Practice. Human ethics approvals were obtained by the University of Melbourne Human Research Ethics Committee (IDs: 1443389.3 and 1443540); the Royal Children's Hospital Human Research Ethics Committee (ID: 24131G); the Mercy Health Human Research Ethics Committee (ID: R14725); and the ARCBS Ethics Committee (ID: 2015#8). All blood donors provided informed written consent for blood donation. Tissues from deceased organ donors were obtained after written informed consent from the next of kin.

### Flow cytometry and cell sorting

Lymphocytes from human thymi, cord blood, lymph nodes (mesenteric and pancreatic), spleen and blood samples were cell surface stained with anti‐CD3‐AF700 (557943)/anti‐CD3‐PE‐CF594 (562280), anti‐CD19‐BV570 (BioLegend, San Diego, CA, USA; 302236), anti‐CD4‐APC‐H7 (560158)/anti‐CD4‐BV650 (563875), anti‐CD8‐BV421 (BioLegend, 301035)/anti‐CD8‐BV650 (563875)/anti‐CD8‐PerCP‐Cy5.5 (565310), anti‐CD27‐BV711 (563167), anti‐CD45RA‐FITC (555488), anti‐PD‐1‐PE‐Cy7 (561272) antibodies (all from BD Biosciences, San Jose, CA, USA), unless otherwise stated with their catalogue number in brackets) and LIVE/DEAD (Aqua, Near‐IR, Molecular Probes, Eugene, OR, USA) or 7‐amino‐actinomycin D (Sigma‐Aldrich, Darmstadt, Germany) on ice for 30 min in PBS. Human PBMCs were sorted for CD27^+^CD45RA^+^ naïve or CD27^+^CD45RA^−^ T_CM_ CD4^+^ and CD8^+^ T cell populations using FACS Aria III (BD Biosciences). Alternatively, cells were fixed and permeablized using the eBioscience Foxp3 staining kit (ThermoFisher Scientific, Waltham, MA, USA) then intracellularly stained with anti‐SATB1‐AF647 (BD Biosciences, 562378) and anti‐GzmB‐AF700 (BD Biosciences, 560213). Samples were acquired using a BD LSR Fortessa (BD Bioscience) and analyzed using FlowJo software (V10.4.2 and 10.5, Treestar, Ashland, OR, USA) and GraphPad Prism (GraphPad Software, La Jolla, CA, USA, V7.02).

### *In vitro* T‐cell stimulation

Sorted naïve CD27^+^CD45RA^+^ and T_CM_ CD27^+^CD45RA^−^ CD4^+^ or CD8^+^ T cells from human PBMCs were labeled with CellTrace violet (ThermoFisher Scientific, Waltham, MA, USA) for 20 min at 37°C, washed, then resuspended in complete RPMI (cRPMI, Gibco, Carlsbad, CA, USA) media (2 mm L‐glutamine, 1 mm sodium pyruvate, 100 μm non‐essential amino acids, 5 mm HEPES buffer, 55 μm 2‐mercaptoethanol, 100 U mL^−1^ penicillin, 100 μg mL^−1^ streptomycin (Gibco), 10% FCS (Bovogen Biologicals, Keilor East, VIC, Australia) at 5 × 10^4^ cells per 1 mL in a 48‐well plate pre‐coated with anti‐CD3‐OKT3 (10 μg mL^−1^), soluble anti‐CD28 (2 μg mL^−1^) (BD Biosciences) and rhIL‐2 (10 U mL^−1^) (BD Biosciences) or rhIL‐2 alone. After 4 days of *in vitro* stimulation, cells were harvested, counted in 0.2% trypan blue (Gibco) using a Neubauer‐improved hemocytometer (Marienfeld, Lauda‐Königshofen, Germany) and stained for flow cytometry.

### Tetramer‐associated magnetic enrichment

HLA‐A*02:01‐GILGFVFTL (A2/M1_58_) and HLA‐A*02:01/GLCTLVAML (A2/GLC) monomer (ImmunoID, University of Melbourne, Melbourne, VIC, Australia) was tetramerized with streptavidin‐phycoerythrin (PE) (BD Biosciences, San Jose, CA, USA). PBMCs from HLA‐A*02:01‐expressing donors were enriched for A2/M1_58_‐ and A2/GLC‐specific CD8^+^ T cells using the A2/M1_58_‐ and A2/GLC‐PE conjugated‐tetramers, respectively, followed by magnetic enrichment as previously described^23^, ^24^, ^32^ using anti‐PE microbeads (Miltenyi Biotec, Bergisch Gladbach, Germany). Enriched, unenriched and flow‐through samples were then stained for flow cytometry.

### Statistical analysis

Statistical analysis was carried out using GraphPad Prism software (GraphPad Software, La Jolla, CA, USA, V7.02). A Student's *t*‐test or one‐way ANOVA was performed for comparison between two or multiple groups, unless stated otherwise. Statistical significance, if present, is described in the figure legend.

## Conflict of Interest

The authors declare no conflict of interest.
