# Supplementary figures and images for: Divergent SATB1 expression across human life span and tissue compartments
Source: Immunol Cell Biol. 2019 Feb 25;97(5):498–511. doi: 10.1111/imcb.12233 (PMC6618325; doi:10.1111/imcb.12233)

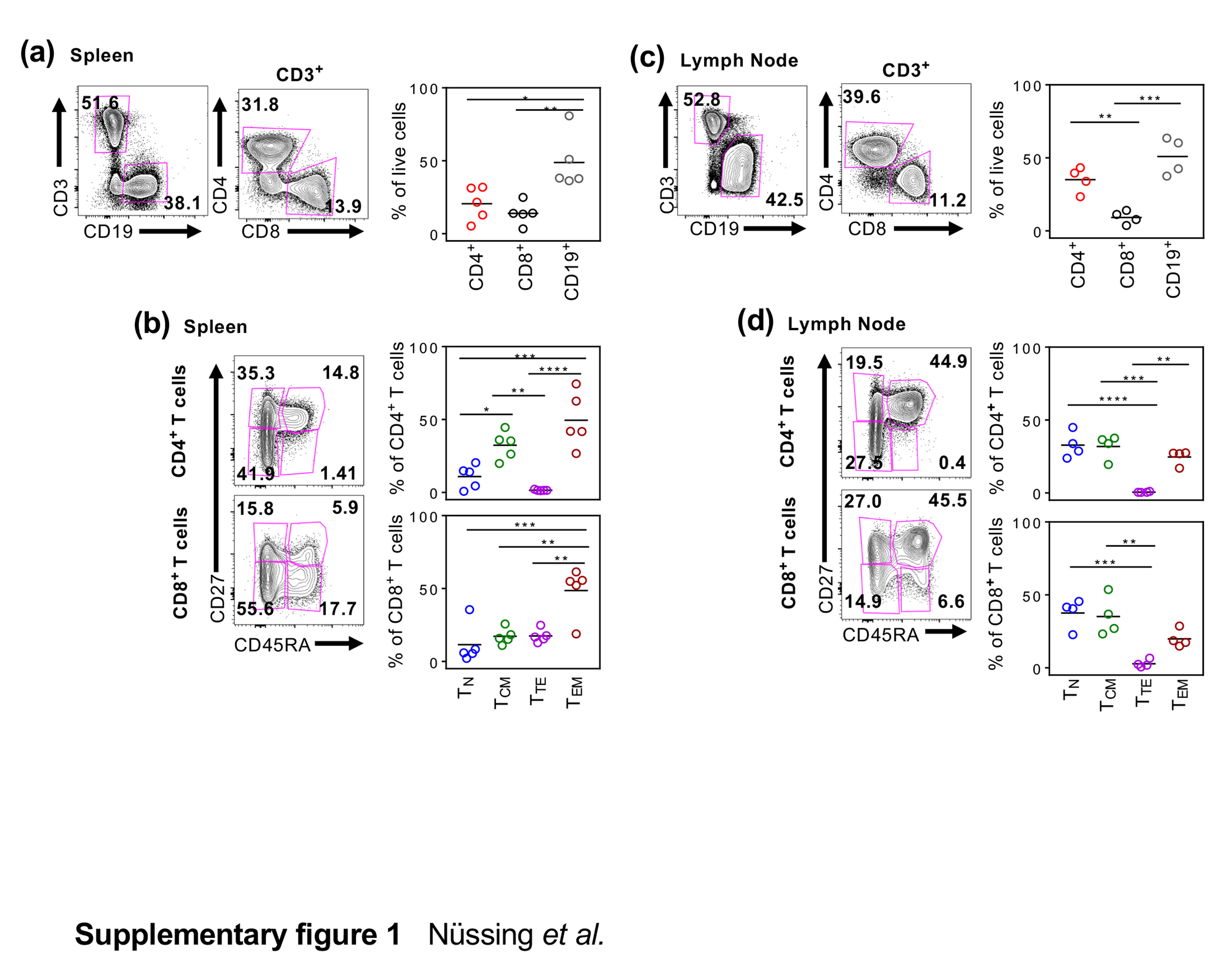

Supplement: Supplementary file 1 [file IMCB-97-498-s001.tif]
