# Supplementary material for: Divergent SATB1 expression across human life span and tissue compartments
Source: Immunol Cell Biol. 2019 Feb 25;97(5):498–511. doi: 10.1111/imcb.12233 (PMC6618325; doi:10.1111/imcb.12233)
Supplement: Supplementary file 2 [file IMCB-97-498-s002.docx]

**SUPPLEMENTARY FILES**

**Supplementary table 1** Demographics of donors used in this study.

| **Organ** | **Donor** | **Age*** | **Gender** | **Figure** |
| --- | --- | --- | --- | --- |
| Thymus | Thy 41  Thy 42  T1  M1  F1 | 6 days  2  8 months  9 months  1.5 | Male  Female  Male  Male  Female | 1 |
| PBMC | BP51  BP54  BP70  BP86  BP87  BP85  BP88  BP100  G94 | 45  60  50  51  58  55  22  45  unknown | Unknown  Unknown  Unknown  Male  Male  Male  Female  Male  unknown | 2  4  2  2  2  4  2/4  2  4 |
| Lymph node | SN208  SN016  SN011  SN025 | 68  28  61  57 | Male  Female  Male  Male | 2 |
| Spleen | SN055  SN208  SN234  SN049  SN339 | 33  68  48  43  51 | Female  Male  Female  Female  Male | 2 |

*Age is in years unless otherwise stated.
